# Pervasive Motor and Balance Difficulties in University Students With Dyslexia

**DOI:** 10.1002/dys.70006

**Published:** 2025-04-25

**Authors:** Martin McPhillips, Helen McNally, Bronagh Taylor, Michail Doumas

**Affiliations:** ^1^ School of Psychology Queen's University Belfast Ireland

**Keywords:** balance, dyslexia, motor difficulties, reflex persistence

## Abstract

Previous research suggests that dyslexic university students are unlikely to experience significant co‐occurring motor or balance difficulties and may represent instances of ‘pure’ dyslexia. However, the motor and balance measures used in previous studies have been limited in scope. The primary aim of the present study was to capture a wider profile of the motor and balance difficulties experienced by dyslexic students. A group of 24 university students with dyslexia were matched on age and IQ to a group of 28 students without dyslexia. Both groups completed standardised tests of reading efficiency, IQ, and attention deficit/hyperactivity disorder (ADHD) symptoms, as well as standardised motor and balance tests and a clinical procedure for primary reflex persistence. The dyslexia group had significant reading efficiency and inattention problems, as well as significantly more difficulties on specific tests of balance and primary reflex persistence. Regression analyses revealed that inattention, balance, and primary reflex persistence were unique predictors of reading efficiency. An individual profile analysis also revealed that 75% of the dyslexic students experienced at least one type of significant motor/balance difficulty (lowest 5 percentiles) relative to their peers. The findings suggest that levels of motor/balance problems in university students with dyslexia have been underestimated in previous research.

## 1 Introduction

Dyslexia may be defined as a ‘pattern of learning difficulties characterized by problems with accurate or fluent word recognition, poor decoding and poor spelling abilities’ (American Psychiatric Association 2013). The predominant causal theory is the phonological deficit theory, which has emerged from a large body of research highlighting the extensive difficulties of struggling readers on a range of phonological tasks (e.g., Peterson and Pennington 2015; Vellutino et al. 2004). However, there is continuing debate whether systematic training in phonics, adopted in some countries over recent decades, has led to improved outcomes in national or international surveys of reading in comparison to other language approaches (e.g., Bowers 2020, 2021; Fletcher et al. 2021; Wyse and Bradbury 2022).

But, irrespective of the method of reading instruction, some children in every country struggle to learn to read, with literacy difficulties persisting across the lifespan. It is, therefore, important that a range of proximal and distal processes that may affect the development of reading skills are explored. While it is generally accepted that children with one developmental difficulty are at risk of other co‐occurring or comorbid developmental difficulties (e.g., D'Souza and Karmiloff‐Smith 2017; Levit‐Binnun et al. 2013), the role of co‐occurrence in the development of reading is less well understood. The co‐occurrence of motor difficulties in high‐achieving young adults with dyslexia (university students), relative to their peer group, is the primary focus of the present study.

There are relatively few studies of motor or balance problems in this population, and a consensus has emerged that such problems occur infrequently and, where present, may be more closely associated with attention deficit hyperactivity/impulsivity disorder (ADHD).

Ramus, Pidgeon, and Frith (2003) hypothesised that students with dyslexia attending university ‘would be least likely to accumulate several types of disorders’, given the potential impact of comorbid issues on longer‐term academic success, and that a university sample would ‘maximise the chances of finding pure cases of the different subtypes of dyslexia’ (p. 844–845). They included 16 students with dyslexia with full‐scale IQ scores > 100 and reading and standard scores < 110 on average, as well as a control group of 17 students. Students with previously diagnosed comorbid disorders were excluded. All participants completed a battery of psychometric, phonological, auditory, visual perception, and cerebellar tests to examine the validity of several competing causal theories of dyslexia, including the cerebellar deficit hypothesis (Nicolson et al. 2001). While there were significant differences between the groups on all the phonological measures, there were no significant differences between the groups on any of the cerebellar tasks, which involved balance, motor coordination and timing. At the individual level, 4 (25%) of the students with dyslexia had an overall composite cerebellar deficit, based on a deviance analysis of 1.65 standard deviations (lowest 5 percentiles) from the control group mean, while 16 (100%) had phonological deficits.

A similar study with 15 Polish university students with dyslexia and 15 controls (Reid et al. 2007) used a range of tests to examine phonological, visual magnocellular, and cerebellar functioning. Using the same deviance procedure as Ramus, Rosen, et al. (2003), Reid et al. found that 87% of the dyslexia group showed phonological deficits and 25% showed a cerebellar deficit relative to the control group. While Ramus, Pidgeon, and Frith (2003), in common with previous studies, interpreted their findings in terms of phonological deficits as the core deficit in dyslexia, with other deficits as non‐causal comorbid markers, Reid et al. highlighted the heterogeneous profiles of the students with dyslexia and suggested that there may be different sub‐types of dyslexia with different underlying causes, including combinations of multiple deficits.

Rochelle et al. (2009) examined the potential role of ADHD symptoms in a sample of 15 university students with dyslexia and 18 controls. Previous work with children suggested that there was a diagnostic overlap between ADHD and dyslexia of 15%–40% and a co‐occurrence of ADHD with motor difficulties of approximately 50% (e.g., Kadesjö and Gillberg 2001; Kaplan et al. 1998). Rochelle et al. (2009) used a balance provocation paradigm and found that the dyslexic students showed significantly more balance perturbations than controls, but that this difference was not significant when inattention and hyperactivity symptoms were considered (6 of the 15 students with dyslexia had ADHD, combined inattention and hyperactivity/impulsivity, scores that warranted further investigation). Although it is difficult to identify the contribution of each of the two significant factors included in the analyses of covariance, it was concluded that cerebellar/balance difficulties in students with dyslexia may be primarily linked to co‐occurring ADHD symptoms. Similarly, in an earlier meta‐analysis involving 17 studies, it was found that differences in balance between dyslexic and control samples were most marked in studies that did not screen for ADHD symptoms (Rochelle and Talcott 2006).

There are, however, major limitations with the cerebellar and motor tests used across previous dyslexia adult (and child) studies, which may explain the variability in results. Commonly, a narrow range of balance or motor tests is used (e.g., Rochelle et al. 2009), or a motor/cerebellar composite score is derived from a small, diverse array of different tasks, where differential performance on specific tasks is minimised (e.g.,Ramus, Pidgeon, and Frith (2003); Reid et al. 2007). Similarly, in a recent study of competing theories of dyslexia, which included fMRI procedures, Danelli et al. (2017) used a single keyboard task to evaluate the relative support for cerebellar theory in 20 dyslexic university students versus 23 controls. They found a significant, reliable hypoactivation in the left occipito‐temporal cortex for the dyslexic group relative to controls when reading but no behavioural or functional imaging cerebellar differences between the groups during the cerebellar task or when reading.

### 1.1 The Present Study

In the present study, we addressed some of the shortcomings of previous research by using a wider range of motor assessment strategies given that the motor system is complex and multi‐faceted and extends beyond cerebellar function or task‐based motor skills. We focused on high‐achieving young adults with a formal diagnosis of dyslexia, who also fulfilled the IQ‐discrepancy definition of dyslexia and were least likely to demonstrate co‐occurrence or comorbidities (Ramus, Rosen, et al. (2003)). The main purpose of the study was to examine how far the co‐occurrence or comorbidity of motor difficulties in university students with dyslexia relative to their peer group may have been underestimated in previous research.

We included a standardised assessment battery of fine motor, gross motor, and balance skills, a standardised clinical protocol for balance problems, and a clinical procedure for assessing the persistence of a primary reflex, the asymmetrical tonic neck reflex (ATNR). The latter procedure was adapted from an earlier study that found a significant association between ATNR persistence and reading attainments in a sample of 515 children (aged 4–8‐years‐old) attending mainstream school in N. Ireland (McPhillips and Jordan‐Black 2007). Children with attention deficits were excluded from this study.

The ATNR was first demonstrated as a brainstem‐mediated response in decerebrate mammals, with afferents originating in the upper cervical spine (Magnus 1926). When the head is turned to one side, there is increased extensor tonus in the limbs on the same side that the head moves towards ‘jaw‐limbs’ and reduced extensor tonus, relaxation, or flexion, in the limbs on the opposing side. Primary reflex tests are commonly used in the paediatric assessment of the neonate, where positive responses are normative (Zafeiriou 2004). However, inhibition or transformation of primary reflexes, including the ATNR, usually occurs in the first year after birth (e.g., Pedroso 2020), and severe persistence beyond early childhood is associated with marked neurodevelopmental and motor difficulties, (e.g., Agarwal and Verma 2012). Importantly, we included a standardised measure of ADHD symptoms and excluded students who had raised combined symptoms (above threshold on both the inattention and hyperactivity/impulsivity subscales) that suggested further exploration for ADHD clinical assessment. As males tend to be over‐represented in studies of dyslexia in children, we included gender as a factor in all group comparisons.

The main aims of the present study were:

1. To assess reading efficiency, attention deficit and hyperactivity/impulsivity symptoms, basic motor function, balance, and ATNR persistence in university students with dyslexia.
2. To examine the relationship between different aspects of motor/balance function and reading efficiency, in the context of potential co‐occurring or comorbid ADHD symptoms.

## 2 Method

### 2.1 Participants

From an original sample of 30 students who volunteered, following a study recruitment notification via the University's disability services, 24 participants with dyslexia (14 male, 10 female) (mean age, 21 years 4 months, SD = 2 years 1 month) were selected to form a dyslexia group. One initial respondent with a full‐scale IQ score of less than 100 and another student undergoing assessment for neurological difficulties were excluded. Four students with raised combined symptoms on an ADHD questionnaire, which suggested further exploration for ADHD clinical assessment, were also excluded. All students with dyslexia had received a formal diagnosis based on assessments by an educational psychologist, either at school or in their first year at university, and none had a comorbid diagnosis of a motor difficulty, such as Developmental coordination disorder (DCD). A control group of 28 participants (13 male, 15 female) (mean age, 21 years 3 months, SD = 1 year 10 months) was recruited via advertisement in the School of Psychology and was matched as closely as possible for age and gender with the dyslexia group. Both groups included undergraduate and postgraduate students; five postgraduates in the dyslexia group and six postgraduates in the control group. Two‐way Analysis of Variance (ANOVA) revealed that there was not a significant difference in age between the groups, *F*(1,48) = 0.03, *p* = 0.876, *η*_p_^2^ *< 0*.01. There was not a significant difference in age according to gender, *F*(1,48) = 0.50, *p* = 0.483, *η*_p_^2^ = 0.01, and there was not a significant interaction between age and gender, *F*(1,48) = 1.34, *p* = 0.253, *η*_p_^2^ *= 0*.03.

### 2.2 Measures

#### 2.2.1 Wechsler Abbreviated Scale of Intelligence (2nd Edition) (WASI‐II) (Wechsler 2011)

The WASI‐II consists of the verbal comprehension index (VCI) (vocabulary and similarities subtests) and the perceptual reasoning index (PRI) (block design and matrix reasoning subtests). The Indices were combined to provide a measure of full‐scale IQ (FSIQ‐4), with a mean of 100 and a standard deviation of 15. The four subtests were administered in approximately 30 min.

#### 2.2.2 Test of Word Reading Efficiency (TOWRE) (Torgeson et al. 1999)

The TOWRE was used to gain a measure of the participants' overall reading level. It involves two subtests that measure sight word reading efficiency (using real words) and phonemic decoding efficiency (using pronounceable nonwords). The scores for each subtest were converted to an overall standardised reading score, with a mean of 100 and a standard deviation of 15. The TOWRE took approximately 5 min to complete.

#### 2.2.3 Conners' Adult ADHD Rating Scales (Long Version) (CAARS) (Conners et al. 1999)

The CAARS was used to provide a measure of the core features of attention deficit/hyperactivity disorder (ADHD) (inattention and hyperactivity/impulsivity) (American Psychiatric Association 2013). The CAARS is a self‐report, 66‐item questionnaire and consists of 8 subscales; the scores for each subscale are converted to *T*‐scores (mean = 50, standard deviatio*n* = 10). Scores above 70 suggest clinically significant difficulties for each subscale. The ADHD Index (overall risk of ADHD) and inattention and hyperactivity/impulsivity subscales were of relevance in the present study.

#### 2.2.4 Movement Assessment Battery for Children (MABC‐2) (Henderson et al. 2007)

The MABC‐2 is a standardised test that is used in clinical practice to provide detailed measures of manual dexterity, aiming and catching skills, as well as static and dynamic balance. The version for 12‐ to 16‐year‐olds (the upper age grouping for this test) was used, as it was thought that 16‐year‐olds would have achieved similar levels of motor skill as young adults attending university. The MABC‐2 consists of 8 subscales that examine 3 composite motor areas: manual dexterity (turning pegs, constructing a triangle, drawing between lines), aiming and catching (catching a ball, aiming a ball at a fixed target on the wall) and balance (balancing on a board with one foot placed in front of the other, maintaining balance while walking backwards on a line and a zig‐zag hopping task). The total test time was approximately 25 min.

Composite standard scores were calculated for manual dexterity, aiming and catching, and balance, as well as total standard motor skills scores (population mean = 10, standard deviation = 3 for each composite and total score).

#### 2.2.5 Sensory Organisation Test (SOT)

The NeuroCom Smart Balance Master system (version 6.1) (Natus Medical Incorporated, USA), which includes a moveable dual force platform and moveable surround, was used to provide a computerised measure of dynamic posturography. The sensory organisation test (SOT) is commonly used in studies of postural control (e.g., Roberts et al. 2021), as well as in clinical settings, and has been normed using a healthy adult population (18–89‐years‐old) (Natus Medical Incorporated 2013). Each participant, in their stockinged feet, was secured in a harness that did not constrain motion while standing on the force platform, according to the indicated markings for each foot displayed on the platform and computer screen. They were instructed to maintain a static upright stance while completing the six standard protocols of the SOT. The protocols have been designed to place demands on the visual, vestibular, and somatosensory systems, which are the three key sensory channels involved in postural control (Peterka 2002). In the first three conditions, the force plates were fixed, and in the final three conditions, they moved in anterior and posterior directions. In the first condition, all sensory information was available. In the second condition, the participant wore a blindfold, eliminating visual information (absent vision). In the third condition, the eyes were open, but the visual surround moved (sway‐referenced vision) thereby introducing inaccurate visual information about body sway. In the final three conditions, the dual force platform moved (sway‐reference support), with eyes open in condition four, eyes closed in condition five (absent vision), and eyes open but moving surround (sway‐referenced support in combination with sway‐reference vision) in condition six. Each condition involved three repeated trials of 20s each, with 1 min of rest between conditions. For all the sway‐referenced conditions, a 1:1 ratio of postural sway to surface and/or visual surround sway in the sagittal plane was used. Postural control was measured as an Equilibrium Score (ES) using the centre of pressure (COP) displacement (degrees of sway). Equilibrium Score has a range from 0 to 100, with larger scores representing less postural sway (better balance) and a score of 100 representing no postural sway. Composite ES values for each of the six conditions were used in the final analysis, as well as an overall total composite score for the six conditions.

**FIGURE 1:**
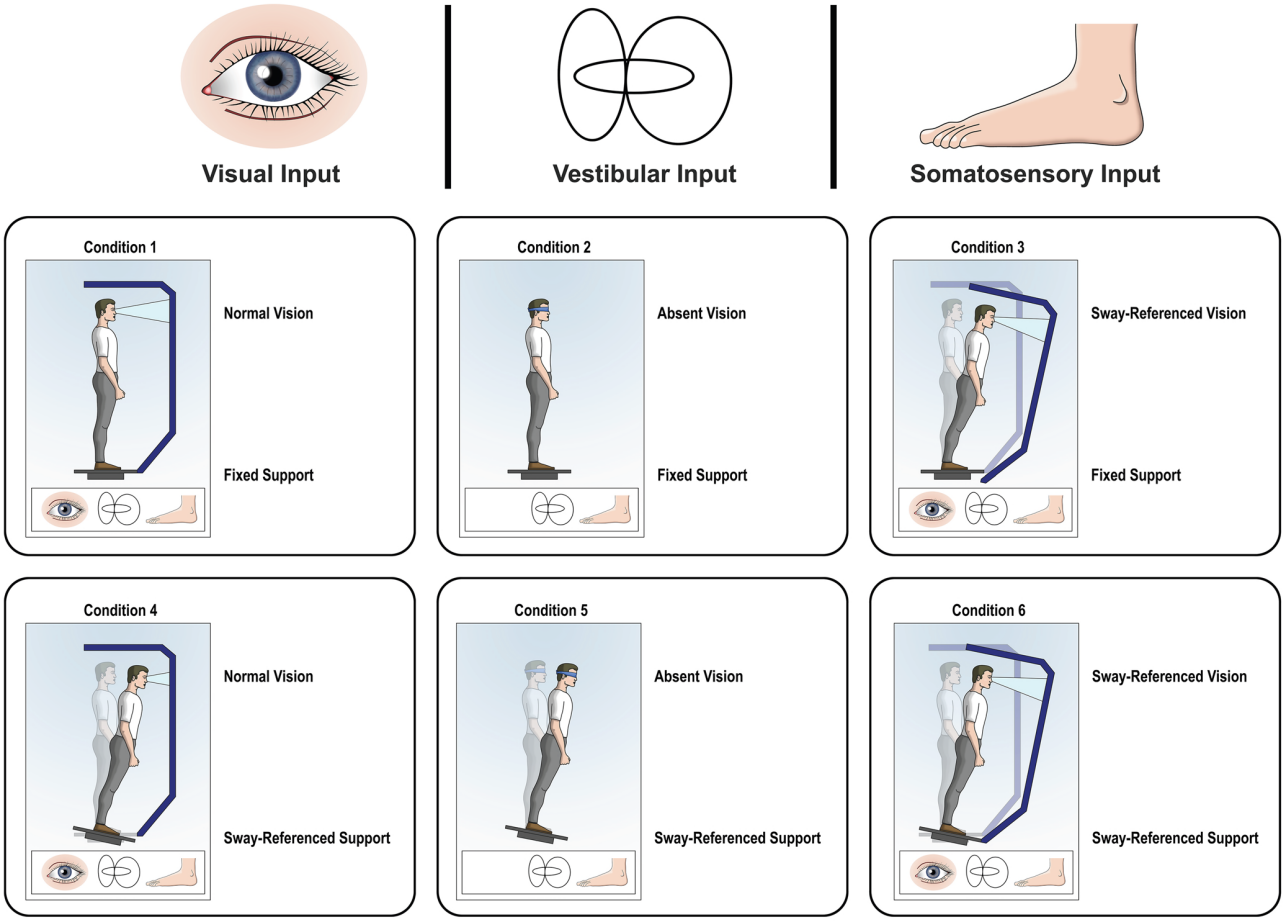
Schematic diagram of the six conditions of the sensory organisation test.

A schematic diagram of the experimental setup is shown in Figure 1.

#### 2.2.6 Adapted Hoff‐Schilder Test

The same clinical protocol from a recent study (see McPhillips et al. 2021), based on the Hoff‐Schilder test (Hoff and Schilder 1927; Morrison 1985), was used to provide a measure of persistence of the asymmetrical tonic neck reflex (ATNR).

‘The Qualisys Motion Capture System (Qualisys, Sweden) was used to provide kinematic measures of arm and head movement. A light display with seven LEDs, which lit up in a sequence moving to either the left side or the right side, was used to provide an external guide for the head movements of the participants. The movements of the wrists and the front of the head were measured using two reflective markers attached to elasticated wristbands worn on the wrists, and one marker attached to the centre of a glasses frame, which rested on the bridge of the nose. Each participant completed two trials with their eyes open with the participant moving their head to follow the light sequence (active movement) followed by two trials with their eyes closed with the experimenter moving their head (passive movement). To avoid order effects, the head turns were counterbalanced for direction of movement. The total extent of movement by the markers was converted to a ratio of total arm movement to total head movement, thus scaling the amplitude of the arm movement with respect to each individual's head rotation’ (McPhillips et al. 2021, 4).

### 2.3 Procedure

Ethical approval for the study was given by the Research Ethics Committee, School of Psychology, XXXXXXXX, in line with the Code of Ethics of the World Medical Association (Declaration of Helsinki). Informed, written consent was provided by each participant before testing began.

Testing was divided into three separate sessions of approximately 30 min each. The participants completed all testing in the Movement Lab at XXXXXXXX. In Session 1, the participants completed the WASI‐II and TOWRE. In Session 2, the participants completed the MABC‐2 and the CAARS, and in Session 3, the participants completed the SOT and the adapted Hoff‐Schilder test. There was a minimum of 3 weeks between each assessment session as the Movement Lab had to be reconfigured for each new set of tasks.

### 2.4 Statistical Analyses

The IBM SPSS 27.0 statistical package (IBM Corporation, New York, USA) was used to analyse the data. Standard MANOVA and ANOVA procedures were used to assess group differences. Simple regression analyses were used initially to identify significant single predictors of reading (total TOWRE scores) and a hierarchical multiple regression analysis was used to evaluate the relative contribution of significant single predictors. Data conformed to the relevant assumptions of normality, multicollinearity, linearity, and homoscedasticity (Field 2013).

## 3 Results

The means (standard deviations) and ANOVA summaries for group differences, including post hoc analyses (Bonferroni), for all cognitive and behavioural measures are shown in Table 1 and for all motor measures in Table 2. Two‐way MANOVAs were used initially for analyses of group differences to reduce the risk of family‐wise error from multiple comparisons. Details for the significant parts of the MANOVA and ANOVA analyses are written out in full below.

**TABLE 1:** Means (and standard deviations) and ANOVA summaries for all cognitive and behavioural measures according to group.

	Dyslexia (D)			Control (C)				
	Male (*n* = 14)	Female (*n* = 10)	Total (*n* = 24)	Male (*n* = 13)	Female (*n* = 15)	Total (*n* = 28)	*F*(1,48)	Post hoc
WASI‐II								
Full IQ	113.1 (4.9)	112.9 (6.9)	113.0 (5.7)	114.2 (5.8)	115.8 (4.6)	115.1 (5.1)	1.69, *p* = 0.199	
VCI	114.2 (9.0)	110.7 (8.7)	112.8 (8.8)	116.5 (7.4)	114.6 (7.5)	115.5 (7.4)	1.87, *p* = 0.178	
PRI	109.1 (6.2)	112.4 (6.5)	110.5 (6.4)	108.6 (7.9)	112.9 (7.0)	110.9 (7.6)	< 0.001, *p* = 0.988	
TOWRE								
Total	75.7 (12.5)	72.3 (15.0)	74.3(13.4)	107.7 (11.2)	103.1 (13.1)	105.2 (12.3)	75.17, *p* < 0.001	D < C
Sight	80.1 (12.7)	75.7 (12.8)	78.3 (12.6)	105.9 (8.1)	96.5 (13.2)	100.9 (11.9)	48.79, *p* < 0.001	D < C
Phonemic	79.3 (9.8)	77.7 (15.8)	78.6 (12.4)	106.7 (15.3)	109.1 (10.9)	108.0 (12.9)	66.27, *p* < 0.001	D < C
CARRS								
ADHD Index	48.4 (8.7)	56.5 (6.4)	51.8 (8.9)	48.7 (9.0)	51.2 (10.1)	50.0 (9.5)	1.01, *p* = 0.321	
Inattention	64.1 (13.3)	65.0 (8.9)	64.5 (11.5)	54.8 (10.7)	54.5 (13.7)	54.6 (12.2)	8.50, *p* = 0.005	D > C
H/I	54.0 (11.8)	54.1 (13.3)	54.0 (12.1)	52.9 (11.3)	48.3 (9.8)	50.4 (10.6)	1.19, *p* = 0.282	

Abbreviations: CAARS = Conners' adult ADHD rating scales (*T*‐scores), TOWRE = test of word reading efficiency (standard scores), WASI‐II = Wechsler abbreviated scale of intelligence (2nd ed.) (standard scores).

**TABLE 2:** Means (and standard deviations) and ANOVA summaries for all motor/balance measures according to group.

		Dyslexia (D)			Control (C)				
		Male	Female	Total	Male	Female	Total	*F*(1,48)	Post hoc
MABC‐2									
Total		8.5 (3.0)	7.4 (2.6)	7.9 (2.8)	10.9 (2.6)	10.1 (1.7)	10.5 (2.2)	12.57, *p* < 0.001	D < C
Composite manual dexterity		8.5 (3.0)	8.2 (2.3)	8.4 (2.6)	9.4 (0.9)	10.5 (2.4)	10.0 (1.9)	5.38, *p* = 0.025	D < C
Turning pegs		8.0 (2.4)	8.5 (3.2)	8.3 (2.8)	8.6 (1.8)	10.3 (2.3)	9.5 (2.2)	1.54, *p* = 0.221	
Triangle with nuts and bolts		8.0 (3.4)	7.0 (1.9)	7.5 (2.7)	7.9 (1.9)	8.3 (2.8)	8.1 (2.4)	0.43, *p* = 0.514	
Drawing trail		7.2 (5.2)	6.1 (5.5)	6.6 (5.3)	10.6 (2.6)	10.1 (3.5)	10.3 (3.1)	9.90, *p* = 0.003	D < C
Composite aim and catch		8.5 (3.0)	8.2 (2.3)	8.4 (2.6)	11.62 (3.6)	8.8 (3.2)	10.1 (3.6)	1.45, *p* = 0.234	
Catching with one hand		10.9 (3.6)	8.4 (3.1)	9.6 (3.5)	11.2 (3.3)	9.3 (3.9)	10.2 (3.7)	0.08, *p* = 0.784	
Throwing at wall target		9.6 (4.7)	6.7 (2.6)	8.1 (4.0)	11.0 (3.6)	8.1 (2.4)	9.5 (3.3)	2.79, *p* = 0.101	
Composite balance		7.7 (2.1)	8.1 (2.9)	7.9 (2.5)	11.2 (2.5)	11.1 (2.6)	11.1 (2.5)	20.70, *p* < 0.001	D < C
Two‐board balance		9.4 (3.3)	9.4 (3.5)	9.4 (3.3)	11.0 (2.4)	12.1 (2.5)	11.6 (2.5)	4.79, *p* = 0.034	D < C
Walk toe‐to‐heel backwards		7.8 (2.6)	7.2 (3.4)	7.5 (3.0)	10.1 (2.7)	10.3 (2.9)	10.2 (2.8)	10.52, *p* = 0.002	D < C
Zig‐zag hopping		8.1 (2.9)	8.9 (2.9)	8.5 (2.9)	11.0 (0.00)	9.4 (3.0)	10.1 (2.3)	5.57, *p* = 0.022	D < C
Sensory Organisation test									
SOT composite	[79.8 (5.7)]	77.6 (7.0)	77.6 (7.9)	77.6 (7.3)	80.9 (4.6)	79.3 (3.7)	80.0 (4.2)	2.95, *p* = 0.092	
SOT 1	[94.0 (2.4)]	94.9 (1.9)	94.1 (2.8)	94.5 (2.4)	95.3 (1.0)	95.3 (1.8)	95.3 (1.5)	3.86, *p* = 0.055	
SOT 2	[92.0 (4.2)]	91.5 (3.1)	90.2 (6.0)	90.8 (4.7)	92.0 (2.1)	92.6 (2.3)	92.3 (2.2)	3.05, *p* = 0.087	
SOT 3	[91.0 (3.0)]	91.6 (3.5)	89.0 (5.5)	90.3 (4.7)	91.5 (2.8)	93.4 (2.0)	92.5 (2.6)	5.01, *p* = 0.030	D < C
SOT 4	[82.0 (4.8)]	86.8 (7.4)	83.9 (9.2)	85.3 (8.3)	89.2 (3.6)	88.0 (3.5)	88.5 (3.5)	3.64, *p* = 0.062	
SOT 5	[69.0 (10.3)]	55.3 (14.0)	62.1 (9.2)	58.7 (12.1)	60.6 (12.4)	62.4 (5.4)	61.6 (9.2)	1.18, *p* = 0.283	
SOT 6	[67.0 (11.5)]	66.3 (15.6)	65.9 (16.3)	66.1 (15.7)	74.2 (7.8)	65.1 (12.6)	69.3 (11.4)	1.41, *p* = 0.240	
Adapted Hoff‐Schilder test									
Eyes open		0.45 (0.21)	0.59 (0.16)	0.52 (0.20)	0.40 (0.10)	0.44 (0.09)	0.42 (0.09)	7.88, *p* = 0.007	D > C
Eyes closed		0.74 (0.26)	0.88 (0.28)	0.81 (0.27)	0.55 (0.12)	0.55 (0.11)	0.55 (0.11)	20.51, *p* < 0.001	D > C

Abbreviations: [] = Manufacturer's condition‐specific norms for 18‐ to 59‐year‐olds. MABC‐2 = movement assessment battery for children (2nd ed.).

### 3.1 Wechsler Abbreviated Scale of Intelligence (2nd Edition) (WASI‐II)

There were no significant group or gender differences, and no interaction effects.

### 3.2 Test of Word Reading Efficiency (TOWRE)

A two‐way ANOVA revealed that the dyslexia group had significantly lower total TOWRE scores than the control group, *F*(1,48) = 75.17, *p* < 0.001, *η*_p_^2^ = 0.61. There were no significant gender or interaction effects.

A two‐way MANOVA revealed that there was a significant difference between the groups on the combined variable of the scores for the two subtests of the TOWRE (sight word reading and phonemic decoding), Wilks' Lambda = 0.38, *F*(2,47) = 37.83, *p* < 0.001, *η*_p_^2^ = 0.62. There were no significant gender or interaction effects. In the follow‐up ANOVAs, the dyslexia group had significantly lower scores on both phonemic decoding and sight word reading than the control group (all *p* < 0.001).

**FIGURE 2:**
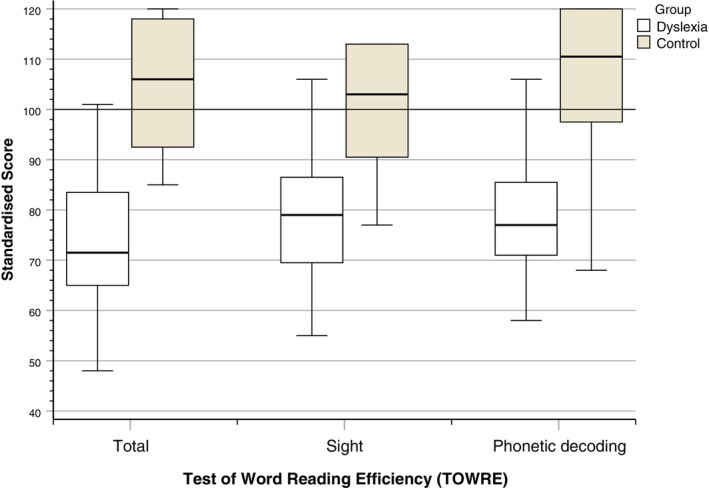
Standardised TOWRE total, sight reading, and phonetic decoding scores according to group.

The distribution of TOWRE total, sight reading, and phonetic decoding standardised scores is illustrated in Figure 2.

### 3.3 Conners' Adult ADHD Rating Scales (CAARS)

A two‐way ANOVA revealed that there was a significant gender effect on ADHD Index scores, *F*(1,48) = 4.63, *p* = 0.036, *η*_p_^2^ *< 0*.09, but not a significant group or interaction effect. Females tended to score higher on the ADHD Index across both groups.

A two‐way MANOVA revealed that there was a significant difference between the groups on the combined variable of the scores for the inattention and hyperactivity/impulsivity scales of the CAARS, Wilks' Lambda = 0.84, *F*(2,47) = 4.63, *p* = 0.015, *η*_p_^2^ *= 0*.17. In the follow‐up ANOVAs, the dyslexia group had significantly higher scores on the inattention scale, *F*(1,48) = 8.50, *p* = 0.005, *η*_p_^2^ *= 0*.15, but not on the hyperactivity/impulsivity scale, *F*(1,48) = 1.19, *p* = 0.282, *η*_p_^2^ *= 0*.02.

**FIGURE 3:**
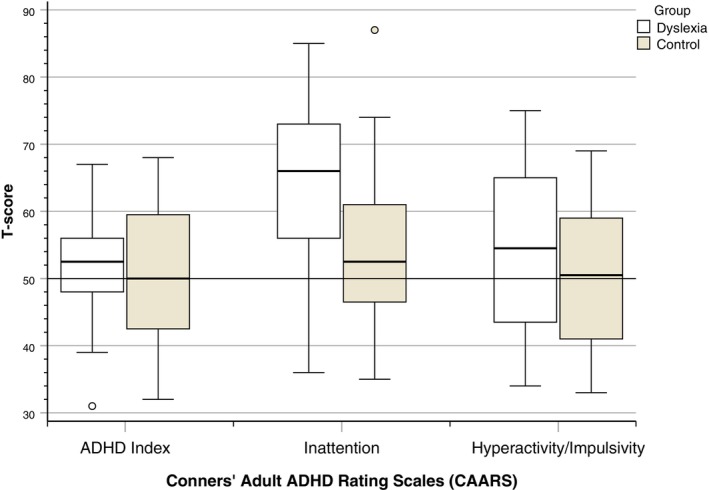
*T*‐scores for CAARS ADHD Index, inattention, and hyperactivity/impulsivity scales according to group.

The distribution of *T*‐scores for the ADHD Index, inattention, and hyperactivity/impulsivity scales according to group is illustrated in Figure 3.

### 3.4 Movement Assessment Battery for Children (2^nd^ Edition) (MABC‐2)

A two‐way ANOVA revealed that the dyslexia group had significantly lower total MABC‐2 scores than the control group, *F*(1,48) = 12.57, *p* < 0.001, *η*_p_^2^ *= 0*.21.

A two‐way ANOVA revealed that the dyslexia group had significantly lower composite manual dexterity scores than the control group, *F*(1,48) = 5.38, *p* = 0.025, *η*_p_^2^ *= 0*.10.

A two‐way ANOVA revealed that the dyslexia group had significantly lower composite balance scores than the control group, *F*(1,48) = 20.70, *p* < 0.001, *η*_p_^2^ *= 0*.30.

There were no significant gender or interaction effects on any of the MABC‐2 composites.

A two‐way MANOVA revealed that there was a significant difference between the groups on the combined variable of the scores for the eight MABC‐2 subtests, Wilks' Lambda = 0.61, *F*(8,41) = 3.29, *p* = 0.005, *η*_p_^2^ *= 0*.391. There was also a significant gender effect, Wilks' Lambda = 0.62, *F*(8,41) = 3.08, *p* = 0.008, *η*_p_^2^ *= 0*.38, but not a significant interaction between group and gender, Wilks' Lambda = 0.92, *F*(8,41) = 0.45, *p* = 0.885, *η*_*p*_^*2*^ *= 0*.08. In the follow‐up ANOVAs, the dyslexia group had significantly lower scores on drawing trail (*p* = 0.003), two‐board balance *(p* = 0.034), walking toe‐to‐heel backwards (*p* = 0.002) and zigzag hopping (*p* = 0.022). Males had significantly higher scores for aiming than females (*p* = 0.003).

**FIGURE 4:**
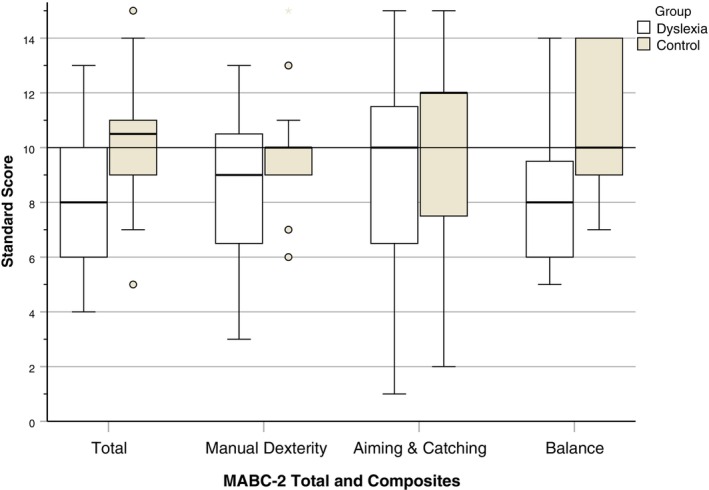
MABC‐2 total and composite standard scores according to group.

The distribution of MABC‐2 total and composite standard scores (based on the standardisation for 16‐year‐olds) is illustrated in Figure 4.

### 3.5 Sensory Organisation Test

There were no significant group or gender differences, and no interaction effects.

### 3.6 Adapted Hoff‐Schilder Test

A two‐way MANOVA revealed that the dyslexia group had significantly higher arm: head ratio scores on the combined variable of the scores for the two conditions of the adapted Hoff‐Schilder test (eyes open and eyes closed), Wilks' Lambda = 0.70, *F*(2,47) = 10.20, *p* < 0.001, *η*_*p*_^*2*^ *= 0*.30. There was also a significant gender effect, Wilks' Lambda = 0.87, *F*(2,47) = 3.57, *p* = 0.036, *η*_p_^2^ *= 0*.13, but there was not a significant interaction between group and gender, Wilks' Lambda = 0.94, *F*(2,47) = 1.61, *p* = 0.211, *η*_p_^2^ *= 0*.06.

In the follow‐up ANOVAs, the dyslexia group had significantly higher arm: head ratio scores in the eyes open (*p* = 0.007) and eyes closed (*p* < 0.001) conditions, with females showing significantly higher ratio scores in the eyes open condition.

**FIGURE 5:**
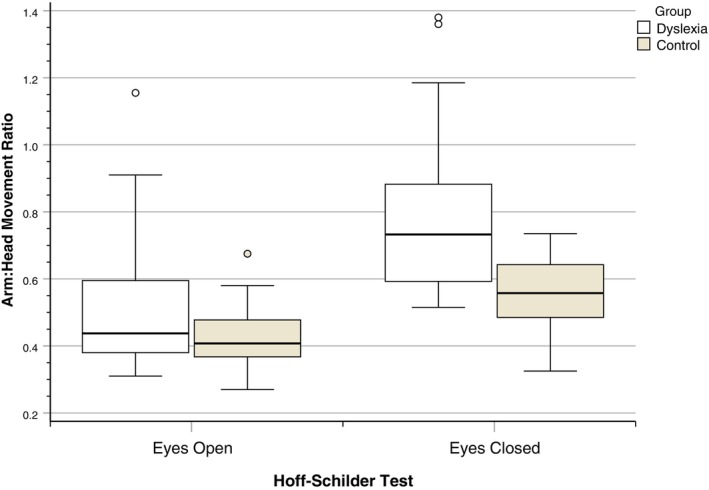
Distribution of arm:head movement ratios on the Hoff‐Schilder test according to group.

The distribution of scores on the Hoff‐Schilder test according to group is illustrated in Figure 5.

### 3.7 Regression

Simple regression analyses showed that inattention, composite balance, and ATNR persistence (adapted Hoff‐Schilder test with eyes closed) were significant individual predictors of reading efficiency (total TOWRE scores). ADHD Index scores (CAARS), hyperactivity/impulsivity, total MABC‐2 scores, drawing trail, composite aiming and catching, and composite SOT scores were not significant predictors of reading efficiency.

A hierarchical multiple regression examined the relative association of the three significant individual predictors (inattention, composite balance (MABC‐2) and ATNR persistence (Hoff‐Schilder)) with reading efficiency (total TOWRE scores). At Step 1, inattention contributed significantly to the model, *F*(1,50) = 7.53, *p* = 0.008, and accounted for 13.1% of the variation in TOWRE scores. When scores for the composite balance (MABC‐2) were added at Step 2, a significant additional 8.4% of the variation in TOWRE scores was explained, *F*(2,51) = 6.71, *p* = 0.003. Finally, the addition of ATNR persistence at Stage 3 explained a significant further 11.4% of the variation in TOWRE scores. In the final model, *F*(3,48) = 7.86, *p* < 0.001, only inattention (*p* = 0.034) and ATNR persistence (*p* = 0.006) were significant, and the linear combination of the 3 predictors accounted for 32.9% of the variation in reading efficiency scores.

A summary table of the 3‐stage hierarchical multiple regression model is shown in Table 3.

**TABLE 3:** Summary of hierarchical multiple regression model for variables predicting reading efficiency (total TOWRE) scores.

	*b*	SE B		*β*	*t*	*p*	*R*	*R* ^2^	∆*R* ^2^
Step 1							0.36	0.13	0.13**
Constant	124.71	12.58			9.91	< 0.001			
Inattention	−0.57	0.21		−0.36	−2.74	0.008			
Step 2							0.46	0.22	0.08*
Constant	99.78	16.27			6.13	< 0.001			
Inattention	−0.49	0.20		−0.31	−2.40	0.020			
MABC‐2 Balance	2.08	0.91		0.30	2.29	0.026			
Step 3							0.57	0.33	0.11**
Constant	126.47	17.84			7.09	< 0.001			
Inattention	−0.42	0.19		−0.26	−2.18	0.034			
MABC‐2 balance	1.14	0.91		0.16	1.25	0.216			
ATNR persistence	−33.08	11.56		−0.37	−2.82	0.006			

* *R* ^2^ change < 0.05.

** *R* ^2^ change < 0.01.

### 3.8 Individual Motor and Balance Profiles

**FIGURE 6:**
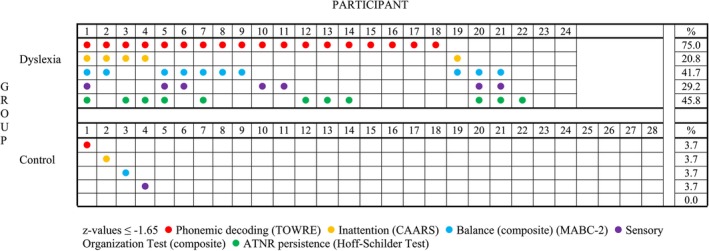
Individual profiles of *z*‐values at or below −1.65 (lowest five percentiles) for each participant according to group.

Z‐scores were calculated for phonemic decoding (TOWRE) scores, inattention scores (CAARS), composite balance (MABC‐2) scores, composite SOT scores, and ATNR persistence (Hoff‐Schilder) scores using the means and standard deviations of the control group for each measure. As in previous studies, z‐scores of −1.65 (the lowest 5 percentiles) were determined for each participant for each measure. The individual profiles of the total sample indicating levels of co‐occurrence are shown in Figure 6.

## 4 Discussion

The first aim of the study was to assess reading efficiency, attention deficit and hyperactivity/impulsivity symptoms, basic motor function, balance and ATNR persistence in university students with dyslexia. Results showed that students with dyslexia experienced significant overall reading difficulties, including significant phonological and sight word reading difficulties, on a standardised measure of reading efficiency in comparison to a control group, who were matched at group level for age and IQ. These results indicate that university students with dyslexia, who may be described as compensated readers (they may have learned to decode text), read significantly slower than their peers, who have not experienced difficulties in learning to read. These findings confirm the persistence of significant language (including phonological) processing difficulties that have been identified as core deficits in dyslexia.

There was not a significant difference in overall levels of ADHD between the groups (students scoring at risk of ADHD were excluded from the study); however, the dyslexic students self‐reported significantly more inattention (but not hyperactivity/impulsivity) symptoms than controls. This highlights the importance of assessing ADHD symptoms when examining the potential role of motor difficulties in dyslexia (e.g., Rochelle and Talcott 2006), given the established co‐occurrence of dyslexia, ADHD, and motor difficulties.

### 4.1 Motor/Balance Function

On a standardised motor assessment (MABC‐2), the students with dyslexia showed significant difficulties on one of the manual dexterity tasks (draw trail) and all three balance tasks relative to controls, with no significant differences between the groups on the aiming and catching tasks. There was a significant gender effect for the aiming and catching tasks, with males, in both groups, performing significantly better than females on both tasks.

No group differences were observed on composite total SOT scores or on the composite scores for the six conditions of the SOT, as measured using computerised dynamic posturography. The means (and standard deviations) for the SOT measures for both groups were very similar to the manufacturer's normative values for standard clinical assessment. While there was evidence of some problems with individual conditions, the overall analyses suggest that balance difficulties may become apparent only under certain task demands. For example, the dynamic posturography measures were all based on the assessment of static balance tasks using force plates, while two of the three balance measures in the MABC‐2 were based on more ecologically valid, dynamic balance tasks. However, it is possible that differential balance responses may have become evident on the SOT if the demands placed on the integration of visual, vestibular, and somatosensory information had been increased using a higher postural sway gain to surface and/or visual surround sway. A previous study assessing postural control in young adults with autism and age and IQ‐matched controls (Doumas et al. 2016) showed that the standard clinical settings (gain = 1) used in the present study did not place sufficient load on the balance system to differentiate more subtle balance differences between the groups. However, group differences emerged when postural sway gain increased (gain = 1.6).

The significant difference between the groups on the Hoff‐Schilder test indicated that young adults with dyslexia may be at increased risk of ATNR persistence. This is an important finding as it suggests that, irrespective of reading remediation or compensation, dyslexic adults are at increased risk of long‐term neurological disturbance from an early reflex system that is most active in the first year of life. This concurs with previous findings with school‐aged children from contrasting socio‐economic backgrounds (McPhillips and Jordan‐Black 2007).

To some extent, the present findings are in line with previous studies that found little or no evidence of significant balance difficulties in university students with dyslexia (Ramus, Rosen, et al. 2003; Reid et al. 2007). The posturography measure used in Ramus et al. did not reveal significant differences between the groups, as was the case in the present study using a clinically normed procedure. Further, the results showed that there were no significant differences between the groups on two fine motor tasks that have been used in previous studies, including bead threading. However, significant motor difficulties were apparent in the present study when other aspects of motor/balance function were included. This suggests that previous studies of young adults (and children) with dyslexia, which have used a relatively limited or narrow range of measures of motor function, may have underestimated the true extent of motor difficulties in young adults (and children) with dyslexia.

The second aim of the study was to examine the relative impact of different aspects of motor/balance function on reading levels, in the context of possible co‐occurring ADHD symptoms. The simple regression models used in the present study aligned with previous findings to some extent, but also emphasised the complexity of motor/balance function. For example, there was evidence of a significant relationship when balance, as measured by the MABC‐2, was isolated as a predictor, but not when measured by the SOT.

Previous work has suggested that the association between motor/balance function and reading difficulties or dyslexia may be better explained by shared levels of comorbid or co‐occurring ADHD‐related symptoms (e.g., Rochelle et al. 2009). The hierarchical regression model showed that, in the present study, inattention symptoms were a significant predictor of reading efficiency; however, balance difficulties (as measured by the MABC‐2) were also a significant unique predictor when added to the model at the second stage. This provides further evidence that the sensitivity of motor/balance measures is important, so that the potential predictive power of balance is not subsumed by the presence of ADHD symptoms in parsimonious models. In the final model, when ATNR persistence was added, balance difficulties were not a significant unique predictor of reading efficiency, while inattention symptoms and ATNR persistence remained significant predictors. Overall, the model showed that the motor/balance difficulties (notably ATNR persistence) experienced by the dyslexic students could not be explained by the presence of co‐occurring inattention symptoms.

The importance of examining the broader profile of motor/balance function was further illustrated by the individual z‐score analyses for each participant. This showed that individuals displayed contrasting motor profiles and that focusing on one dimension of motor function may not capture the breadth of motor/balance difficulties experienced by dyslexic adults. In the present study, 75% of the dyslexic students displayed marked difficulties with z‐scores in the bottom five percentiles on at least one of the three motor/balance measures used. This contrasts with the level of difficulties (25%) reported in previous work (Ramus, Pidgeon, and Frith 2003; Reid et al. 2007), which used a limited range of assessment approaches and collapsed motor/balance function into one overall motor/balance composite. In other words, even in a relatively ‘pure’ sample of dyslexic adults (who fulfilled the discrepancy definition), co‐occurring motor/balance difficulties may be much more pervasive than previously thought.

The importance of early motor function in later cognitive development is increasingly recognised. For example, data from the Millenium Cohort study in the UK revealed how early fine and gross motor skills (at 9‐months‐old) were predictive of later cognitive outcomes at 5‐years‐old (Schoon et al. 2010), and some recent smaller studies have also emphasised the importance of fine and gross motor skills in early readiness for schooling. For example, Suggate et al. (2019) found that fine motor skills, as measured by the three manual dexterity tasks in the MABC‐2, contributed unique variance in the early development of reading skills, independent of related cognitive and language factors, and Kamphorst et al. (2021), using the complete MABC‐2 battery, found that motor skills, particularly balance, were associated with four distinct school readiness profiles.

However, while it is generally acknowledged that motor/balance difficulties may co‐occur with dyslexia to some extent, it is argued that they should be viewed as comorbid symptoms and not causal, as they are not exclusive to dyslexia (e.g., Goswami 2015; Ramus, Pidgeon, et al. 2003). For example, some adults (and children) with motor/balance difficulties do not experience dyslexia and not all dyslexics experience motor/balance difficulties. The implication is that only factors that are associated exclusively with one developmental problem should be considered causal. A contrasting view suggests that motor/balance difficulties, which occur across different atypical patterns of development, should be viewed as neurodevelopmental markers of vulnerable brain states, or atypical brain development, that place the child at risk of one or more developmental disorders (e.g., Dewey and Bernier 2016; Gillberg 2010; Levit‐Binnun et al. 2013).

From a neuroconstructivist perspective, studies with compensated adults with childhood dyslexia may be particularly problematic in determining the validity of different theories of dyslexia at the neural level, as adults may show ‘domain‐specific’ neural activations that may not reflect earlier ‘domain general’ developmental processes (e.g., D'Souza and Karmiloff‐Smith 2017). Longitudinal studies are required to determine the potential role of different behavioural and neural systems in the emergence of specific neurodevelopmental disorders over time, including dyslexia. The present study highlights the importance of task sensitivity in such work.

### 4.2 Limitations and Future Work

The sample size in the present study is relatively small, although larger than in comparative studies with this population. The cross‐sectional design is a major limitation, and it is not possible to determine how far reading and motor developmental pathways may intersect without longitudinal designs. The present study included an explicit marker of neurodevelopmental delay (ATNR persistence), which was present at a significant level in 46% of the dyslexic students. Ultimately, intervention studies are also key to understanding the importance of different aspects of motor function (and language factors) in early reading development, and while there is some tentative evidence that interventions that target reducing ATNR persistence may improve reading and writing skills in children with dyslexia (e.g., McPhillips et al. 2000), larger‐scale, school‐based studies are required.

Another limitation concerns the differentiation of the measures used in the present study. Although the dyslexic students were compensated readers, only one age‐appropriate reading test with two dimensions was used to assess phonemic and sight word reading levels, and it would have been interesting to have incorporated some other measures of reading and language function, such as comprehension skills.

### 4.3 Conclusions

Our findings suggest that levels of motor/balance problems in university students with dyslexia, relative to their peers, may have been underestimated in previous behavioural studies, and these difficulties may not be entirely explained in terms of co‐occurring or comorbid ADHD symptoms. In particular, the persistence of a brainstem‐mediated early reflex was evident at a significant level in the dyslexic adults and was also a significant independent predictor of reading efficiency. Further work is needed to understand how underlying mechanisms associated with motor/balance difficulties and reflex persistence may impact the development of the reading network over time, and interventions that address underlying motor/balance and reflex persistence issues, which could be used to complement language‐based interventions, should be evaluated.

## Ethics Statement

The study was approved by the Research Ethics Committee, School of Psychology, Queen's University, Belfast, in line with the Code of Ethics of the World Medical Association (Declaration of Helsinki).

## Consent

Informed, written consent was provided by each participant before testing began.

## Conflicts of Interest

The authors declare no conflicts of interest.

## Data Availability

The data that support the findings of this study are available from the corresponding author upon reasonable request.
